# Etymologia: *Streptococcus*

**DOI:** 10.3201/eid2211.ET2211

**Published:** 2016-11

**Keywords:** etymologia, Streptococcus, Streptococcus pyogenes, Streptococcus pneumoniae, bacteria, streptococci, streptococcal diseases, erysipelas, Theodor Billroth

## *Streptococcus* [strepʺto-kokʹəs] 

From the Greek *streptos* (“chain”) + *kokkos* (“berry”), streptococcal diseases have been known since at least the 4th century bce when Hippocrates described erysipelas (Greek for “red skin”). The genus *Streptococcus* (Figure) was named by Austrian surgeon Theodor Billroth, who in 1874 described “small organisms as found in either isolated or arranged in pairs, sometimes in chains” in cases of erysipelas or wound infections. Over subsequent decades, as microscopy and staining techniques improved, many different researchers characterized the bacteria now known as *Streptococcus pyogenes* (Lancefield group A β-hemolytic streptococcus), *S. pneumoniae*, and other species.

**Figure:**
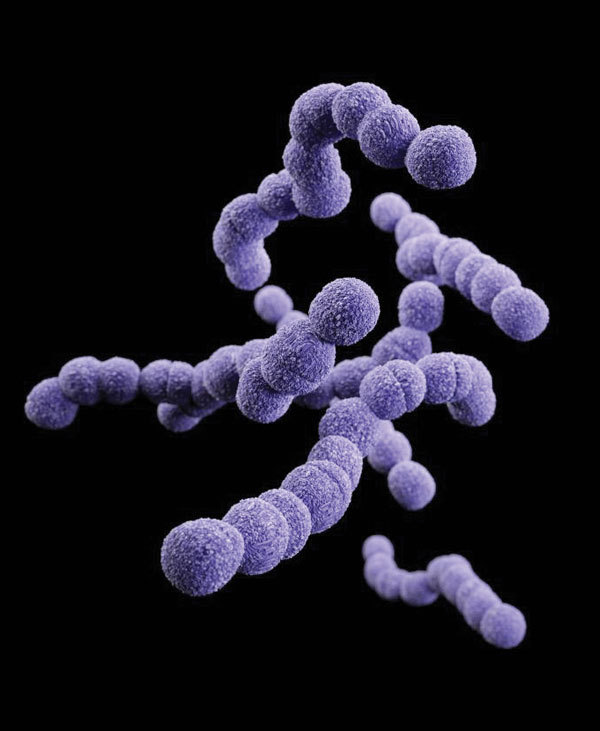
Clindamycin-resistant group B *Streptococcus.* Photo: Centers for Disease Control and Prevention.
